# Relation Between Perinatal Depressive Symptoms, Harm Avoidance, and a History of Major Depressive Disorder: A Cohort Study of Pregnant Women in Japan

**DOI:** 10.3389/fpsyt.2019.00515

**Published:** 2019-07-26

**Authors:** Chika Kubota, Toshiya Inada, Tomoko Shiino, Masahiko Ando, Branko Aleksic, Aya Yamauchi, Maya Sato, Masako Ohara, Satomi Murase, Mako Morikawa, Yukako Nakamura, Takashi Okada, Setsuko Goto, Atsuko Kanai, Norio Ozaki

**Affiliations:** ^1^Department of Psychiatry, National Center Hospital of Neurology and Psychiatry, National Center of Neurology and Psychiatry, Kodaira, Japan; ^2^Department of Psychiatry, Nagoya University Graduate School of Medicine, Nagoya, Japan; ^3^Department of Psychiatry and Psychobiology, Nagoya University Graduate School of Medicine, Nagoya, Japan; ^4^Center for Advanced Medicine and Clinical Research, Nagoya University Graduate School of Medicine, Nagoya, Japan; ^5^Liaison Medical Marunouchi, Nagoya, Japan; ^6^Goto Setsuko Ladies Clinic, Nagoya, Japan; ^7^Graduate School of Education and Human Development, Nagoya University, Nagoya, Japan

**Keywords:** major depressive disorder, harm avoidance, postpartum depression, psychometrics, psychiatric status rating scales

## Abstract

**Introduction:** The relationship between perinatal depressive symptoms, harm avoidance (HA), and a history of major depressive disorder (MDD) was examined in a prospective cohort study.

**Methods:** This study was conducted from May 1, 2011, to December 31, 2016. A history of MDD was evaluated using the Inventory to Diagnose Depression, Lifetime version during pregnancy. Depressive state and HA were evaluated during pregnancy and at 1 month postnatal using the Edinburgh Postnatal Depression Scale (EPDS) and Temperament and Character Inventory, respectively. The relationship between these variances was examined using structural equation modeling.

**Results:** A total of 338 participants with complete data were included in the present study. Pregnant women with compared with those without a history of MDD were observed to have a significantly higher intensity of HA and more severe depressive symptoms in both the prenatal and postnatal periods. A history of MDD affected the severity of depressive symptoms [standardized path coefficient (SPC) = 0.25, *p* < 0.001] and the intensity of HA during pregnancy (SPC = 0.36, *p* < 0.001). The intensity of HA during pregnancy affected that at 1 month postnatal (SPC = 0.78, *p* < 0.001), while the severity of depressive symptoms as assessed by the EPDS during pregnancy affected that at 1 month postnatal (SPC = 0.41, *p* < 0.001). The SPC for perinatal HA to postnatal depressive symptoms (SPC = 0.13, *p* = 0.014) was significant and higher than that for perinatal depressive symptoms to postnatal HA (SPC = 0.06, *p* = 0.087).

**Conclusion:** The present results suggest that early intervention in pregnant women with a history of MDD or a high intensity of HA is important to prevent postnatal depressive symptoms.

## Introduction

The global prevalence of postnatal depression (PND) has been estimated to be approximately 10% (1–4). PND influences not only the quality of life of mothers, but also child development and the child-rearing environment (5–7). Therefore, the prevention of PND is important. Especially in Japan, the perinatal suicide rate from 2005 to 2014 in Tokyo has been estimated to be 8.7 per 100,000 births (8). In addition, 13 of 40 women were reported to commit suicide after birth because of the development of PND (8). To prevent maternal death, investigating the predictors of PND onset in Japan is considered an urgent issue.

In a study investigating the relationship between depression and temperament, a strong association was reported between the severity of depression and the intensity of harm avoidance (HA) (9). HA is one of the personality traits and a concept that consists of the following four factors: anticipatory worry, fear of uncertainty, shyness with strangers, and fatigability and asthenia (10). In a previous study, Andriola et al. investigated the relationship between HA and depressive symptoms during the perinatal period in 65 pregnant woman and found that a high intensity of HA was a predictor of prenatal depression (11). In a study of 601 pregnant Japanese women, Minatani et al. reported that the severity of prenatal depressive symptoms was predicted by a negative response toward the current pregnancy, low self-directedness, a high intensity of HA, persistence, and self-transcendence (12). Josefsson also reported that HA was higher in women with PND (13). Based on the results of a meta-analysis of the predictors of PND, a history of major depressive disorder (MDD) was reported as a risk factor (14, 15). It has also been reported that an accumulated number of depressive episodes may result in increased HA (16). However, the causal relationships between perinatal depressive symptoms, HA, and a history of MDD have not been adequately investigated.

In a depressive state, the proportion of behavioral avoidance from aversive environments is high (17). In recent years, it has been reported that avoidance behavior leads to depression (18). From this point of view, behavioral activation has recently come to be adopted in psychotherapy (19).

If the intensity of prenatal HA affected the prevalence of postnatal depressive symptoms, psychological approaches to HA for pregnant women would be useful for PND prevention. Alternatively, if the prevalence of prenatal depressive symptoms affected the intensity of postnatal HA, screening for prenatal depression with subsequent early treatment would be valid. In addition, a history of MDD may affect the intensity of perinatal HA and depressive symptoms.

Our research group has been conducting a prospective cohort study of PND in Nagoya since 2004. In our previous study, the intensity of HA was compared between a control and a PND group using data from 2004 to 2010 (20). Although no significant differences were found in the intensity of HA between the control and PND groups during pregnancy, the intensity of HA was higher in the PND than in the control group at 1 month postnatal. Therefore, HA is considered to be state dependent and not to be a risk factor for PND. However, in our previous study, participants with temporary gestational and continuous depressive symptoms were excluded, and we did not investigate the participants’ history of MDD. Moreover, the correlation between depressive symptoms and HA was examined, so the causal relationships between these variables remain unclear.

In the present prospective cohort study, we have investigated participants’ history of depression since 2011 using the Inventory to Diagnose Depression, Lifetime version (IDDL). The purposes of the present study are to clarify the following points using data obtained since 2011:

1) A history of MDD will affect the intensity of HA and the severity of depressive symptoms during the perinatal period.
2) The severity of prenatal depressive symptoms will affect the intensity of postnatal HA, and the intensity of prenatal HA will affect the severity of postnatal depressive symptoms. Another purpose of the present study is to clarify the influence of prenatal depressive symptoms and HA on the postnatal period.

## Methods

### Study Design

This prospective cohort study on perinatal depressive symptoms was conducted in Nagoya, Japan. The data in the present study, including the IDDL in the assessment, were extracted from May 2011 to the end of December 2016.

### Participants

The eligibility criteria were as follows: 1) pregnant women aged 20 years or older, 2) ability to answer questionnaires written in Japanese, and 3) received an obstetrical examination at one of following four hospitals: one general hospital (Nagoya Teishin Hospital), two obstetrics and gynecology hospitals (Kaseki Hospital and Royal Bell Clinic), and one university hospital (Nagoya University Hospital). Participants were recruited from the attendants of a maternity class (childbirth education class) or the outpatient clinic.

### Ethical Considerations

This study was approved by the ethics committee of Nagoya University Hospital. All procedures were conducted in accordance with the Declaration of Helsinki. Written and verbal informed consent for participation was obtained from all participants.

### Measurements

The psychosocial backgrounds of the participants were evaluated during pregnancy and at 1 month *postpartum* using a self-administered questionnaire.

#### A History of Major Depressive Disorder (MDD)

A history of MDD was assessed using the IDDL during pregnancy. The IDDL is a self-administered questionnaire that was developed by Zimmerman and Coryell in 1987 to assess the history of MDD on the basis of the third version of the Diagnostic and Statistical Manual of Mental Disorders (DSM-III) (21). The IDDL is composed of 22 items scored on a five-point Likert scale. All 22 items on the IDDL are classified into nine symptoms (two major and seven other symptoms) according to the DSM-III criteria for MDD. The criteria for diagnosing a history of MDD were as follows: (1) having five or more of the nine symptoms and (2) these symptoms contain one or more of the two major symptoms. The sensitivity and specificity of the IDDL are reported to be 74% and 93%, respectively (21). The Japanese version of the IDDL was validated by Uehara et al., and the sensitivity and specificity were reported to be 83% and 97%, respectively (22).

#### Perinatal Depressive Symptoms

Perinatal depressive symptoms were rated using the Edinburgh Postnatal Depression Scale (EPDS) during pregnancy and at 1 month postnatal. The EPDS was developed by Cox et al. in 1987 to screen for PND (23). It is a self-administered questionnaire composed of 10 items scored on a four-point Likert scale. The total score can range from 0 to 30 points. The Japanese version of the EPDS was validated by Okano et al. in 1996 (24). We also confirmed the reliability and validity of the Japanese version of the EPDS for perinatal women using data from our prospective cohort study in Nagoya and identified its factor structure (25, 26).

#### Harm Avoidance (HA)

HA was rated using the Temperament and Character Inventory (TCI) (27). The TCI is a self-administered questionnaire composed of 125 items that is based on Cloninger’s seven-dimensional model (four temperament and three character dimensions) (28). Twenty of the 125 items on the TCI are on HA, and the total score ranges from 0 to 20 points. The Japanese version of the TCI was also validated by Kijima et al. in 1996 (29).

### Statistical Analyses

**Figure 1:**
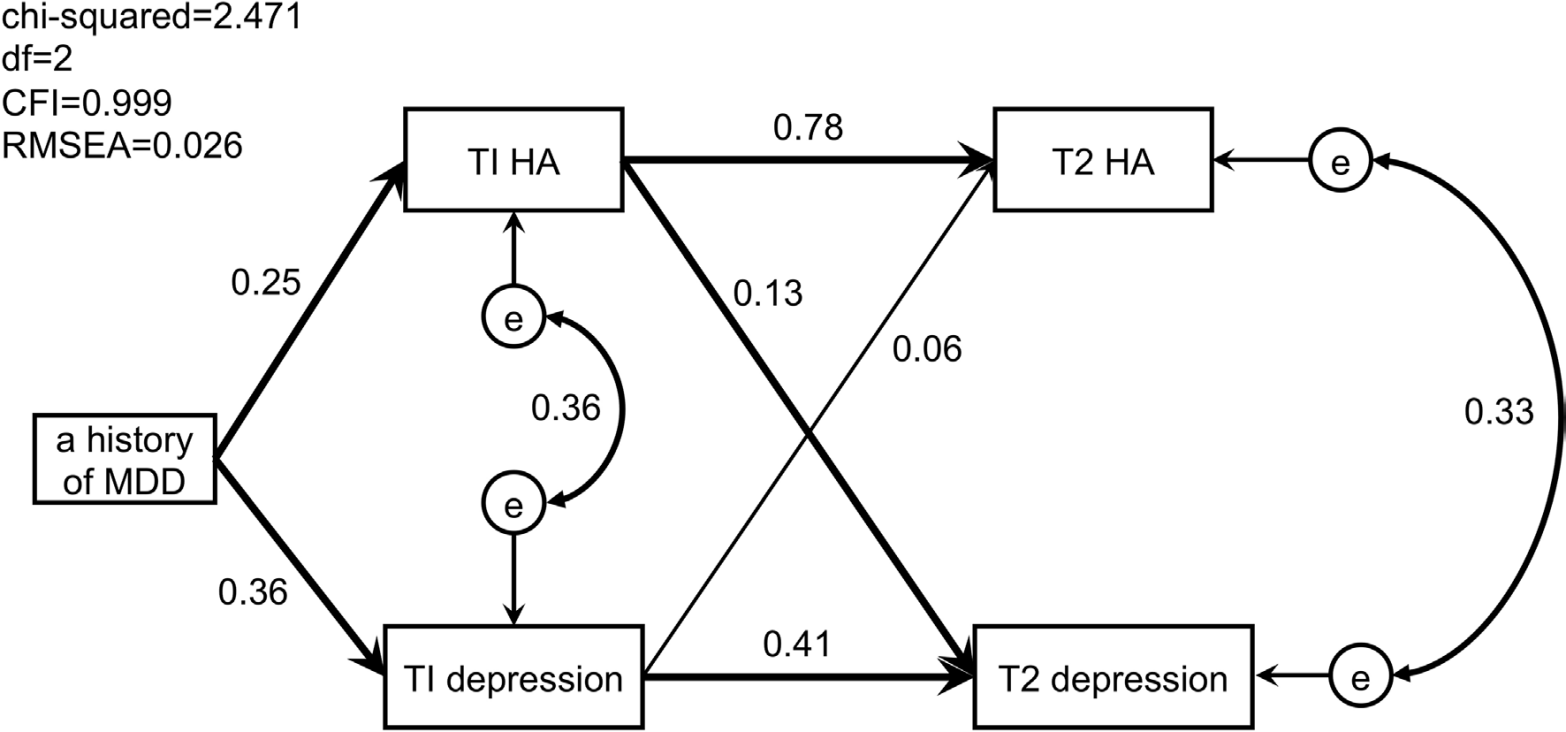
Path model of perinatal depressive symptoms, harm avoidance, and a history of major depressive disorder. Significant paths are in bold. T1, during pregnancy; T2, at 1 month postpartum; MDD, major depressive disorder; HA, harm avoidance; e, error.

The mean, standard deviation (SD), and Pearson’s correlation coefficient (CC) were calculated for each variable. The proportion of pregnant women with a history of MDD was calculated using the IDDL. Each variable was compared in regard to the presence or absence of a history of MDD based on the IDDL. In addition, the proportion of pregnant women suspected of having perinatal depression was calculated using the EPDS during pregnancy and at 1 month postnatal. Correlations were defined as poor at <0.2, fair at <0.4, good at <0.6, very good at <0.75, and excellent at <1 (30). The relationships between the following variables were investigated using structural equation modeling (SEM): the presence or absence of a history of MDD based on the IDDL, total score on the EPDS during pregnancy and at 1 month postnatal, and total score for HA on the TCI during pregnancy and at 1 month postnatal. An element that could not be explained by the measurement variables was defined as an error (“e” in**** **Figure 1**). The listwise method was used to handle missing values.

The following fit indexes were calculated: the chi-square normalized by degrees of freedom (CMIN/df), the comparative fit index (CFI) (31), the root mean square error of approximation (RMSEA) (32), and Akaike’s information criterion (AIC) (33). The goodness of fit of the path model was defined as good with CMIN/df <1, CFI >0.97, and RMSEA <0.05 (31, 32), and as acceptable with CMIN/df <3, CFI >0.95, and RMSEA <0.08 (31, 32). AIC estimates the relative quality of the models for the same data, and a lower score is better as an index to evaluate each model (33). All statistical analyses were performed using IBM SPSS (version 25.0; IBM Japan) and IBM SPSS Amos (version 25.0; IBM Japan).

## Results

### Characteristics of the Participants

A total of 455 participants voluntarily participated in the present study. Statistical analysis was conducted on all 338 participants who completed all of the items. All participants were evaluated during pregnancy (22.7 weeks gestation, SD 6.3 weeks) and at 1 month postnatal (32.8 days postnatal, SD 10.5 days). The mean age of the participants was 32.6 years (SD 4.7 years). Regarding parity, the rates of nulliparas, primiparas, those who had given birth twice, and those who had given birth three times or more were 82.9%, 13.7%, 3.0%, and 0.3%, respectively. The ratio of participants who scored 13 points or higher on the EPDS was 6.5% during pregnancy and 11.2% at 1 month postnatal, respectively. The proportion of participants who had a history of MDD based on the IDDL was 30.7%. The mean total EPDS scores were 4.99 (SD 4.63) during pregnancy and 5.88 (SD 5.30) at 1 month postnatal, the mean HA scores were 12.06 (SD 4.43) during pregnancy and 12.49 (SD 4.26) at 1 month postnatal, and the mean IDDL score was 30.86 (SD 16.56).

**Table 1** shows a comparison of the variables between pregnant women with and without a history of MDD. Pregnant women with a history of MDD had a significantly higher intensity of HA and more severe depressive symptoms in both the prenatal and postnatal periods compared with those without.

**Table 1:** Comparison of the variables between pregnant women with and those without a history of MDD.

	History of MDD (−)		History of MDD (+)		
	M	SD	M	SD	*t* value
Age	32.53	4.74	32.65	4.74	−0.211
T1 HA	11.32	4.22	13.71	4.46	−4.736***
T1 EPDS total score	3.88	3.74	7.47	5.41	−6.164***
T2 HA	11.85	4.16	13.90	4.15	−4.184***
T2 EPDS total score	5.00	4.53	7.84	6.29	−4.165***

T1, during pregnancy; T2, 1 month postpartum; MDD, major depressive disorder; HA, harm avoidance; EPDS, the Edinburgh Postnatal Depression Scale. ***p < 0.001.

### Correlations Between the Variables

**Table 2** shows the means and SDs of all the variables and Pearson’s CCs between two of the following five variables: 1) a history of MDD, 2) HA during pregnancy (T1 HA), 3) depressive symptoms during pregnancy (T1 EPDS), 4) HA at 1 month postnatal (T2 HA), and 5) depressive symptoms at 1 month postnatal (T2 EPDS). The skewness and kurtosis of each variable were both <3, as follows: a history of MDD (0.822, –1.332), T1 HA (–0.320, –0.644), T1 EPDS (1.363, 2.451), T2 HA (–0.402, –0.541), and T2 EPDS (1.165, 1.373). Therefore, Pearson’s CCs were calculated assuming a normal distribution. Regarding the CCs between two of these five variables, the following contents were revealed: (1) a strong CC (*r* = 0.804, *p* < 0.01) was observed between the intensity of HA during pregnancy and that at 1 month postnatal. Contrary to this finding, the CC between the intensity of HA (T1 and T2) and the severity of depressive symptoms (T1 and T2) was relatively low (*r* = 0.300–0.433), 2) the CC between a history of MDD and the severity of depressive symptoms during pregnancy was moderately significant (*r* = 0.359, *p* < 0.01), 3) a moderate significant correlation (*r* = 0.466, *p* < 0.01) was observed between depressive symptoms during pregnancy and at 1 month postnatal, and 4) the CC between a history of depression and the intensity of HA was 0.250 (*p* < 0.01) during pregnancy and 0.223 (*p* < 0.01) at 1 month postnatal.

**Table 2:** Correlations between the variables.

	History of MDD	T1 HA	T1 EPDS	T2 HA	T2 EPDS
History of MDD	1.000				
T1 HA	0.250**	1.000			
T1 EPDS	0.359**	0.416**	1.000		
T2 HA	0.223**	0.804**	0.385**	1.000	
T2 EPDS	0.248**	0.300**	0.466**	0.433**	1.000

T1, during pregnancy; T2, 1 month postpartum; MDD, major depressive disorder; HA, harm avoidance; EPDS, the Edinburgh Postnatal Depression Scale. **p < 0.01.

### Path Model

The path model of a history of MDD in association with perinatal depressive symptoms and HA is shown in **Figure 1**. The following points were clarified: 1) a history of MDD predicted the severity of antenatal depressive symptoms (SPC = 0.36, *p* < 0.001) and the intensity of antenatal HA (SPC = 0.25, *p* < 0.001); 2) the intensity of antenatal HA predicted the severity of postnatal depressive symptoms (SPC = 0.13, *p* = 0.014) and the intensity of postnatal HA (SPC = 0.78, *p* < 0.001); 3) the severity of antenatal depressive symptoms predicted that of postnatal depressive symptoms (SPC = 0.41, *p* < 0.001), but not the intensity of postnatal HA (SPC = 0.06, *p* = 0.087); and 4) errors in the intensity of HA and the severity of depressive symptoms were observed both during pregnancy (CC = 0.36) and at 1 month postnatal (CC = 0.33).

## Discussion

In this prospective cohort study that followed up 338 participants, the following three causal relationships between perinatal depressive symptoms, HA, and a history of MDD were clarified. First, a history of MDD predicted the antenatal severity of depressive symptoms and the antenatal intensity of HA during pregnancy. Second, the intensity of antenatal HA predicted the severity of postnatal depressive symptoms and the intensity of postnatal HA. Third, the severity of antenatal depressive symptoms predicted postnatal depressive symptoms, but not the intensity of postnatal HA.

From the previous results of our ongoing cohort study, Furumura et al. hypothesized that the intensity of HA may be state dependent (20); however, according to our present results, the intensity of antenatal HA predicted the severity of postnatal depressive symptoms (SPC = 0.13). A possible reason for the methodological difference between the previous and current studies is that the participants in the previous study were limited to healthy antenatal women. As a result, 81 of 460 participants were excluded from the analysis (20).

As shown in **Table 1**, a significant difference was observed between pregnant women with and without a history of MDD for the following variables: HA during pregnancy, EPDS total score during pregnancy, HA at 1 month postnatal, and EPDS total score at 1 month postnatal. The present results suggest that a history of MDD may predict the severity of depressive symptoms and the intensity of HA during the perinatal period. To prevent PND, psychological care from the early stage of pregnancy may be important for women with a history of MDD.

Compared with the stronger correlation observed between antenatal and postnatal HA (*r* = 0.804, *p* < 0.01) than that between antenatal and postnatal depressive symptoms (*r* = 0.466, *p* < 0.01), the correlations between perinatal HA and depressive symptoms were relatively weaker during pregnancy (*r* = 0.416, *p* < 0.01) and at 1 month *postpartum* (*r* = 0.433, *p* < 0.01). Thus, the intensity of HA was not affected by other variables before or after delivery, which supports the hypothesis that temperament is an inherent personality trait (34). A stronger correlation between a history of MDD and the severity of depressive symptoms was observed during pregnancy compared with at 1 month postnatal. Although the IDDL assesses the history of MDD, its precise period of development cannot be confirmed. Having depressive symptoms continuously before pregnancy may contribute to the higher correlation between a history of MDD and the severity of antenatal depressive symptoms. However, there is a possibility that other risk factors influenced PND independent of a history of MDD. In future studies, it will be necessary to consider other risk factors as well.

In the present study, the proportion of participants who had a history of MDD diagnosed using the IDDL was 30.2%. This rate among pregnant Japanese women is higher than that reported in previous studies. For example, Zimmerman et al. reported a rate of about 15% among women in the U.S. (21), while Uehara et al. reported a rate of about 15% among 93 Japanese women (35), and Sakado et al. reported a rate of about 13% among another 126 Japanese women (36). These differences may derive from different backgrounds in regard to whether the participants were pregnant women or healthy women are general.

A number of limitations should be considered when interpreting our results. First, the existence of the aforementioned selective bias needs to be taken in account. In the present study, questionnaire responses were received by post, so it is possible that those who were not interested in health and mental illness did not respond. In addition, the target facilities are limited to four hospitals in Nagoya city, one of which is a university hospital that admits pregnant women with many complications. In addition, since a large number of pregnant women were attending the maternity class, we did not count the number of pregnant women who did not participate in the present study after explaining its aims and methods. Second, in regard to the sample size, the number of participants in the present study satisfied the proposed criteria that required at least 200 cases to be included in the analysis for SEM (37). Further research with a larger sample size and more variables will be required to conduct more appropriate SEM. Third, SEM is an analysis method for verifying an arbitrary path model based on a researcher’s hypothesis (38). Since a better model or more influential variables may exist, further research using larger number of participants or variables is needed to investigate the appropriateness of our model. Fourth, the psychiatric variables used in the present study were all evaluated based on a self-rated questionnaire, and no direct interviews were conducted with psychiatrists; these might allow more precise and reliable psychiatric information to be obtained.

In conclusion, the present results suggest that early intervention is important to prevent PND in pregnant women with a history of MDD. To help prevent PND, it may be indispensable to clarify the temperament of the woman before childbirth, as the severity of HA during pregnancy can predict PND.

## Data Availability

The datasets generated for this study are available on request to the corresponding author.

## Ethics Statement

This study was carried out in accordance with the recommendations of the ethics committee of Nagoya University Hospital. The protocol was approved by the ethics committee of Nagoya University Hospital.

## Author Contributions

SM, SG, AK, and NO conceived and designed the experiments. CK, YN, AY, TS, MM, MO, MS, and TO performed the experiments. CK, TI, MA, and NO conducted the statistical analysis. CK, TI, BA, and NO wrote the paper. All authors contributed to and approved the final manuscript for submission.

## Funding

Funding for this study was provided by research grants from the Ministry of Education, Culture, Sports, Science and Technology of Japan; the Ministry of Health, Labour and Welfare of Japan; The Academic Frontier Project for Private Universities, Comparative Cognitive Science Institutes, Meijo University; the Core Research for Evolutional Science and Technology; Intramural Research Grant (21B-2) for Neurological and Psychiatric Disorders from the National Center of Neurology and Psychiatry; the Specific Research Fund 2012 for East Japan Great Earthquake Revival by The New Technology Development Foundation and the Japan Agency for Medical Research and Development; and Research and Development Grants for Comprehensive Research for Persons with Disabilities from Japan Agency for Medical Research and Development (AMED) under Grant No. JP18dk0307077.

## Conflict of interest Statement

The authors declare that the research was conducted in the absence of any commercial or financial relationships that could be construed as a potential conflict of interest.
